# Combined effect of hydrotherapy and transcranial direct-current stimulation on children with cerebral palsy: A protocol for a randomized controlled trial

**DOI:** 10.1097/MD.0000000000027962

**Published:** 2021-12-10

**Authors:** Xiao-Liang Chen, Li-Ping Yu, Ying Zhu, Tie-Yan Wang, Jing Han, Xiao-Yan Chen, Jia-He Zhang, Jia-Li Huang, Xiao-Ling Qian, Bo Wang

**Affiliations:** a Department of Pediatrics, The Second Affiliated Hospital of Qiqihar Medical University, Qiqihar, Heilongjiang, China; b Department of Nursing, Rehabilitation Center Hospital of Gansu Province, Lanzhou, Gansu, China; c The Second Clinical Medical College, Lanzhou University, Lanzhou, Gansu, China; d Department of Neurosurgery, The First Affiliated Hospital of Xi’an Jiaotong University, Xi’an, Shaanxi, China; e School of Public Health, Lanzhou University, Lanzhou, Gansu, China; f Department of Neurology, The Second Hospital of Lanzhou University, Lanzhou, Gansu, China.

**Keywords:** cerebral palsy, children, hydrotherapy, transcranial direct current stimulation

## Abstract

**Background:**

Cerebral palsy (CP) is a neurodevelopmental disorder caused by a brain injury resulting in poor coordination and motor control deficits, which is one of the most common physical disabilities in children. CP brings a heavy burden on families and society and becomes a significant public health issue. In recent years, hydrotherapy, and transcranial direct current stimulation (tDCS) as a physical therapy for CP is developing rapidly. When hydrotherapy and tDCS are used to treat separately, it has positive therapeutic effect in children with CP. The development of new therapies in combination with physical rehabilitation approaches is critical to optimize functional outcomes. tDCS has attracted interest in this context, because of significant functional improvements have been demonstrated in individuals with brain injuries after a short period of cerebral stimulation. Since the onset of this work, tDCS has been used in combination with constraint-induced therapy, virtual reality therapy to potentiate the treatment effect. Up to now, there are no studies on the effect of a combined application of hydrotherapy and tDCS in children with CP. We will conduct a 2-arm parallel clinical trial to investigate the effect of a combined application of tDCS and hydrotherapy.

**Methods and analysis:**

This study is an outcome assessor and data analyst-blinded, randomized, controlled superiority trial during the period from October 2021 to December 2023. CP patients meeting the inclusion criteria will be allocated in a 1:1 ratio into the treatment group (hydrotherapy plus tDCS), or the control group (treatment as usual). All participants will receive 30 sessions of treatment over 10 weeks. The primary outcomes will be the difference in the Gross Motor Function Assessment and Pediatric Balance Scale during rest and activity. The secondary outcomes will be the difference in adverse effects between the control and treatment groups.

**Conclusions:**

This study aims to estimate the efficacy of a combined application of tDCS and hydrotherapy in patients with CP.

**Trial Registration:**

This study protocol was registered in Chinese ClinicalTrials.gov, ID: ChiCTR2100047946.

## 1 Introduction

Cerebral palsy (CP) is a neurodevelopmental disorder caused by a brain injury resulting in poor coordination and motor control deficits, which is one of the most widespread physical deformity in children.^[1]^ This disorder commonly occurs in 2.11 cases per 1000 live births and are widely recognized for causing dystonia, motor, and movement dysfunction, and intellectual disability.^[2,3]^ A previous meta-analysis demonstrated that the prevalence of CP among children aged 0 to 6 years in China is 0.23%.^[4]^ The European surveillance of CP group described the motor type of CP as spastic, dyskinetic (dystonia, chorea, and athetosis), ataxic and mixed types involving the mentioned types.^[5]^ Spastic cerebral palsy is the most common type of this movement disorder, which occurs in more than 80% in children.^[6]^ CP is usually diagnosed in childhood, but its course can last into adolescence, adulthood, and has different severity. There are evidences that children with CP have a higher rate of deterioration of physical and mental function and a higher risk for secondary diseases.^[3,7]^ It is reported that the cost of care for each child is estimated at $921,000 in the US.^[8]^ CP brings a heavy burden on families and society and becomes a significant public health issue.^[9]^

The conventional treatment for children with CP is made up of medical treatment, physical therapy (PT), occupational therapy, psychotherapy, surgical therapy, virtual reality technology, hyperbaric oxygen therapy, stem cell transplantation, and speech therapy.^[10–12]^ Almost all these treatments are aimed at improving patient activity and functional participation, however, none of them can completely cure CP. In recent years, PT as a treatment for CP is developing rapidly, which is a special treatment method based on physical factors such as sound, electricity, light, force, water, magnetism, and thermal power. Owing to the risks of neurosurgery and side effects of pharmacological intervention, PT becomes the central part of rehabilitation for children with CP.^[13,14]^ Among them, hydrotherapy promotes children's voluntary and passive movement in the water through the resistance, buoyancy, static pressure, and warming effects of water, which is beneficial to improve human blood circulation and relax the muscles of the whole body, thereby promoting the alleviation of muscle spasm and the reduction of muscle tension, and then improves the children's balance ability, expands the joint range of motion.^[15]^ Numerous studies have pointed out that the potential of hydrotherapy programs have significant benefits for children with CP.^[15,16]^ The result of a 10-week aquatic exercise training program is effective in the functional rehabilitation of children with spastic CP.^[17]^ A previous systematic review published in 2017 suggested that evidence on aquatic interventions for children with CP is limited since only 2 used randomized control trial design, and the results were mixed.^[18]^ Another systematic review published in 2021 showed that hydrotherapy can help improve the motor function and activities of children and adolescents with CP, and may improve their quality of life, which included 9 randomized controlled trials (RCTs).^[19]^

Noninvasive brain stimulation is one of PT which is under active investigation in childhood neurology and psychiatry, especially in disorders where focal cortical over- or under-activation is part of the pathophysiology.^[20]^ Among them, 2 are developing rapidly, they are transcranial magnetic stimulation and transcranial direct current stimulation (tDCS). tDCS constant electrical currents are conducted to the brain via scalp electrodes, which is undergoing investigation as a credible treatment for a series of neuropsychiatric diseases. This technique shares a capacity to modulate regional cortical excitability, and well-tolerated by children and adults.^[21,22]^ Now, active tDCS research is pushed in part by a favorable tDCS safety profile, the low cost of tDCS stimulator, and by fairly repeatable effects on the cortex, where (coarse) exposure to cathodal current brings about cortical suppression and exposure to anodal current brings about cortical activation.^[23]^ tDCS has been tested as a treatment for several pediatric neurologic conditions.^[20,22]^ Several RCTs also reported a beneficial effect of tDCS in patients with autism spectrum disorder,^[24]^ attention-deficit/hyperactivity disorder,^[25]^ epilepsy^[26]^ and CP. The finding from a meta-analysis suggested that tDCS can improve the static balance in children with CP at follow-up and have a positive effect on gait velocity.^[27]^ The heterogeneity of participant characteristics, dosing parameters, and outcome indicators makes it difficult to integrate citations and directly apply them to clinical practice. Caution must be taken when using this summary as an implementation guide.^[27]^ The Commenter of this meta-analysis suggested that researchers could reduce heterogeneity, increase sample size, avoid unnecessary repetition of additional pilot studies as positive results have been confirmed, and establish a focus on immediate and longer-term participant-centered efficacy outcomes while closely monitoring safety, so that future research will be more meaningful to clinicians.^[28]^

To sum up, when hydrotherapy and tDCS are used to treat separately, it has positive therapeutic effect in children with CP. The development of new therapies in combination with physical rehabilitation approaches is critical to optimize functional outcomes. tDCS has attracted interest in this context, as significant functional improvements have been demonstrated in individuals with brain injuries after a short period of cerebral stimulation. Since the onset of this work, tDCS has been used in combination with constraint-induced therapy, gait and mobility training, virtual reality therapy to potentiate the treatment effect.^[29–31]^ Up to now, there are no studies on the effect of a combined application of hydrotherapy and tDCS in children with CP. We will conduct a 2-arm parallel clinical trial to investigate the effect of a combined application of tDCS and hydrotherapy. We hypothesized that tDCS combined concurrently with hydrotherapy would result in the greater and longer-lasting improvement in motor function.

## 2 Methods and analysis

### 2.1 Study design

**Figure 1:**
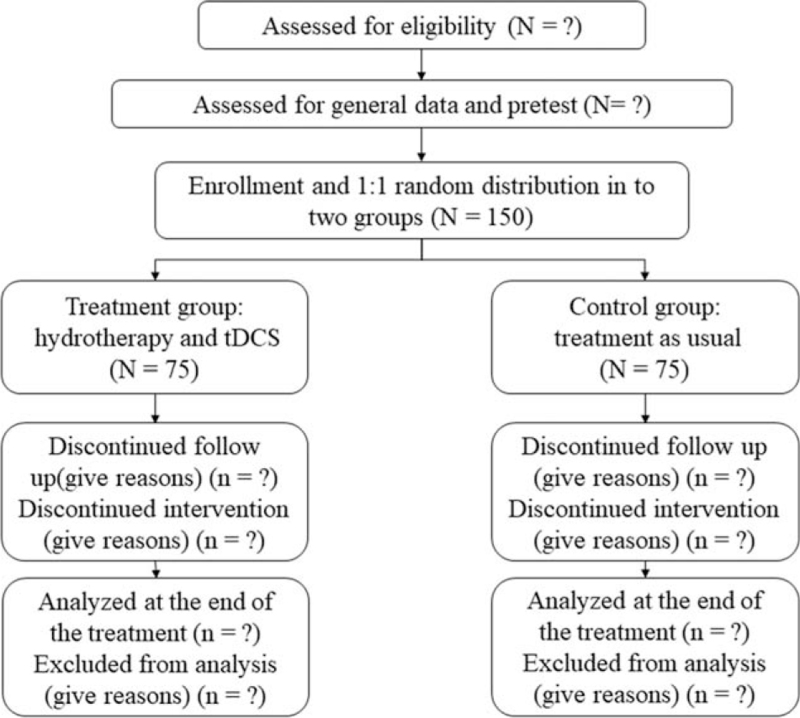
CONSORT flowchart demonstrating the progress of 2 groups through the randomized controlled trial phases. tDCS = transcranial direct current stimulation.

This study is an outcome assessor and data analyst-blinded, randomized, controlled superiority trial and will be conducted at the Second Affiliated Hospital of Qiqihar Medical University, from October 2021 to December 2023. CP patients who meet the inclusion criteria will be allocated in a 1:1 ratio into the treatment group (hydrotherapy plus tDCS, n = 75, expected), or the control group (treatment as usual, n = 75, expected). All participants will receive 30 sessions of treatment over 10 weeks. The study design is presented in Figure 1.

### 2.2 Participants

Participants meeting the following inclusion criteria will be included: CP patients between 4 and 14 years old, CP was diagnosed according to the Subspecialty Group of Rehabilitation of Chinese Medical Association,^[32]^ voluntary participation and informed consent signed. Participants with any of the following exclusion criteria will be excluded: visual, auditory, and mental disorders affecting the results assessment; children with epilepsy who are uncontrolled with medication; skin problems such as open wounds or active infection; receiving botulinum toxin injections or surgery no earlier than 6 months before project start; participation in another clinical trial; unconscious, behaving strangely or uncontrollably, and are prone to medical accidents during treatment.

### 2.3 Sample size

The necessary sample size has been estimated based on study of Whitehead et al,^[33]^ which estimated the minimum sample size of a pilot randomized trial with a continuous outcome variable. A previous RCT protocol about the effect of bilateral anodal tDCS vs treadmill training on brain activities of children with CP also used this Sample size estimation method. In our study, the Gross Motor Function Measure (GMFM) score which is a continuous outcome variable, is regarded as a primary outcome. The result is that 150 children with CP will undergo intervention or control group and will be assessed just once during pretest assessment to consider their results as a reference value for later comparisons. However, there is a lack of official pediatric reference values in GMFM of study on the combination of hydrotherapy and tDCS. Compared with the results of a meta-analysis, the sample size of our study is larger than that of each RCT on tDCS.^[34]^ Therefore, generalization of reference values from this study should be made with caution.

### 2.4 Randomization

The participants will be assessed for the first time, pre-intervention, and then randomly assigned to one of the 2 groups: combination treatment group (treatment with hydrotherapy and tDCS), control group (treatment as usual). The randomization sequence will be distributed by numerically sequenced and sealed opaque envelopes. This process will be completed by an independent researcher.^[35]^ A second independent number performed the randomization. A series of numbered, sealed, opaque envelopes will be used to ensure concealed allocation. Each envelope will contain a card stipulating to which group the child will be allocated. This study will use the blind statistical analysis, where the statistical data will be analyzed by statisticians with unclear grouping and its meaning.

### 2.5 Evaluation and follow-up

Two experienced physical therapists who blind to which group each child belongs will evaluate the both group in the evaluation procedures. Four evaluations will be conducted:

First evaluation: 1 week before intervention;
Second evaluation: 1 week after the last intervention;
Third evaluation: 4 weeks after the last intervention;
Fourth assessment: 12 weeks after the last intervention.
These evaluations will be conducted on 2 nonconsecutive days.

### 2.6 Outcome measures

We will evaluate the CP children using questionnaires at the beginning of the treatment, within 1 week after 10 weeks of therapy, 4 weeks, and 12 weeks after the last intervention. Descriptive measures and characteristics, such as age, gender, body weight, type of CP and the outcomes were recorded for all study participants. All patient related data will be safely collected and stored. Only researchers related to the study will be allowed to access to the trial dataset. The primary outcomes of this study are Gross Motor Function Assessment and Pediatric Balance Scale (PBS) between the treatment group and the control group. The secondary outcomes are the difference in Pediatric Evaluation Disability Inventory (PEDI), adverse effects between the control and treatment groups.

#### 2.6.1 Primary outcomes measures

Gross Motor Function Measure 88 (GMFM-88): The GMFM-88 will be used to detect gross motor function change in this study.^[36–38]^ The GMFM-88 is made up of 5 dimensions that can be analyzed separately or combined to produce a total GMFM-88 score. The 5 dimensions are as follows: lying and rolling, sitting, crawling and kneeling, standing, and walking, running, and jumping. All items are scored on a 4-point scale, the higher the score, the better the function.^[36–38]^ The treating therapist, in collaboration with parents and children, will choose Gross motor goal dimensions while considering the age of children and gross motor function classification system level. All evaluation process will be recorded and later scored by a senior physiotherapist who is blind to group allocation. The results will be reported as percentage scores.

PBS: The same blinded researcher will administer the PBS before and after the intervention.^[39–41]^ To assess balance, the PBS will be used, which is composed of 14 items. The items assess the functional activities which children could perform to function within the home, school, or community safely and independently. This scale has also been tested for children with CP and has good test–retest and interrater reliability when used assess participation of school-age children with mild to moderate movement disorder.^[39–41]^

#### 2.6.2 Secondary outcome measures

The (PEDI, the PEDI is a questionnaire that quantitatively measures functional performance. This questionnaire includes 3 areas: self-care, mobility, and social function. The self-care part contains food, personal hygiene, toilet use, clothing, and toilet control. The functional items of mobility offered information about transfers, walking indoors and outdoors, and use of stairs. The social function part mirrors matters related to communication, problem solving, interaction with partner, amongst others. We will calculated total scores for each scale in each area, where each item has a score of 0 (the child is unable to play an activity) or 1 (the activity is part of the child's repertoire), and the sum of the items generates the score for each field.^[42,43]^

Adverse effects. We set this standard to refer to the specific details of previous study.^[35]^ Children with CP will be asked to answer on a 3-point scale: I did not feel unwell at any moment during the session; I felt unwell at some points during the session; I felt unwell throughout the session. The specific unwell are as follows: tingling sensation, burning sensation, headache, pain in the region where the electrodes were positioned, drowsiness, and mood swings. When participants pointed that they have specific adverse effect, in other words, the response is “2” or “3”, they will be instructed to quantify the intensity of perception (1, weak; 2, moderate; 3, strong; or 4, unbearable).

### 2.7 Intervention

#### 2.7.1 Control group: usual care

Both groups of participants will continue to receive “usual care” during the 10-week intervention. Usual care refers to any therapeutic treatment or service that the child would normally receive outside of the intervention study. The researcher will evaluate children's condition, we expected that most participants in this study will not access additional therapy as possible.

#### 2.7.2 Treatment group: hydrotherapy and tDCS

Our intervention plan is based on Akinola et al's^[17]^ research on hydrotherapy and studies about tDCS.^[30,44,45]^

The participants in the treatment group will receive a treatment protocol which is about specific plans and measures during treatment. Participants will receive exercise training in water, 2 times a week for 10 weeks, with the exercise area fully immersed in water. The water temperature will be controlled between 28°C and 31°C throughout the whole duration of intervention. Two physical therapists will be involved in the treatment of each participants in a treatment session. The exercise program contains 2 categories of exercises as follows.

Exercise 1 (manual passive stretching). This will include passively moving the joint involved in the spastic muscle group away from the direction of the main function and maintain this position for 60 seconds, where the muscle group is fully extended. This process will be repeated 5 times for each part, with a total duration of 5 minutes.

Exercise 2 (functional training). All children will receive 4 levels of functional training according to their dysfunction levels, and each level of training lasted 15 minutes. The 4 levels are the following:

Level 1: 2-point kneeling exercise training
Level 2: Sitting education/training
Level 3: Standing education/training
Level 4: Walking education/training

Two neurologists with ample experience in noninvasive cerebral stimulation will oversee the evaluations for the indication of tDCS. tDCS will be performed during the intervention sessions, it is reported that tDCS can promote behavioral changes through building a neural network that is conducive to the environment. Our study will employee the tDCS Transcranial Stimulation equipment producted by Soterix Medical Inc. The device works by 2 5 × 5 cm^2^ nonmetallic sponge surface electrodes immersed in saline solution. The anodal electrode will be in the dominant brain hemisphere area over C3, following the internationally standardized 10 to 20 electroencephalogram system, corresponding to the primary motor cortex, and the cathode will be located in the contralateral supraorbital area.^[45]^ No stimulation will be provided throughout the rest of the session, which is a valid control procedure in studies involving tDCS. The current will be applied to the primary motor cortex for 20 minutes in the middle of each training session. The device has a button and the operator can control the intensity of the current. The stimulation will increase from 0 to 1 mA and gradually reduced in the final 10 seconds. The tDCS will be repeated once a day, 3 times a week, a total of 30 treatments

We initially plan to carry out tDCS on Mondays, Wednesdays, and Fridays, and hydrotherapy on Thursdays and Sundays.

### 2.8 Statistical analysis

An experienced statistician will conduct the data analysis. When necessary, intention to treat analysis will be used with the data from the previous evaluation repeated to substitute missing data. We will use the Kolmogorov-Smirnov test to demonstrate normal data distribution. Thus, Parametric data will be presented as mean (standard deviation) as well as nonparametric data will be presented as median (inter-quartile interval). The effect size will be calculated by the difference between means of the pre-intervention and post-intervention evaluations and will be presented with the respective 95% confidence interval. The independent *t* test and chi-square tests will be applied to assess and compare the baseline patient characteristics between the treatment and the control groups. Either 2-way ANOVA or the Kruskal-Wallis test will be used for the statistical analysis of the effects of control group or treatment group for parametric and nonparametric variables, respectively. The Bonferroni correction for multiple comparisons will be employed as the post hoc test. Statistical significance will be considered at a *P* value of <.05. The data will be organized and tabulated using the Statistical Package for the Social Sciences (SPSS) version 19.0 (IBM, Armonk, NY, USA).

## 3 Discussion

According to incomplete statistics, there were about 5.3 million patients with CP in China in 2018, and it is increasing at a rate of about 50,000 per year. The investigation report of children with CP in 12 provinces in China pointed out that the incidence of CP in children aged 1 to 6 years was 2.48‰, and the most common type of CP was spastic CP, which accounted for 58.86% of children with CP. Children with spastic CP are unable to stand, sit up and walk normally due to rigid extension reflexes, overexcited reflexes, and abnormal muscle stiffness, which is the main cause of severe motor disability in children.^[46]^ Children with CP are a huge group, and their treatment and rehabilitation is an important task in our country. Rehabilitation therapy plays a key role in the management of CP in children. It can help children with CP to maximize their potential for physical independence and health, and minimize the impact of physical injury. At present, the rehabilitation treatment of CP is mainly based on PT, including exercise therapy, hydrotherapy, occupational therapy, and tDCS. Among the outcome indicators such as motor function and balance function, it has been proven that hydrotherapy and tDCS are effective for children with CP. This study protocol describes a RCT study design which will test the comparative effectiveness of combining hydrotherapy and tDCS. The outcome evaluations will include analyses of GMFM-88, PBS. If the combination of hydrotherapy and tDCS is proven to be superior to the other in some of these aspects, this evidence would permit professionals to recommend the ideal PT for patients with CP, which will minimize their incapacity and optimize their rehabilitation. It is hoped that findings from this research will be published and disseminated in an internationally recognized, peer-reviewed journal.

## 4 Conclusion

In conclusion, this protocol describes an outcome assessor and data analyst-blinded, randomized, controlled superiority trial that aims to investigate the efficacy of a combined application of tDCS and hydrotherapy in patients with CP. The results will ascertain whether tDCS combined concurrently with hydrotherapy would result in the greater and longer-lasting improvement in motor function. The findings will be of great significance for clinical practice about treating CP.

## 5 Ethics and dissemination

The trial will be commenced after ethical approval has been obtained from the Second Affiliated Hospital of Qiqihar Medical University. All study-related procedures will be performed only after obtaining written informed consent from children and their parents. Patients’ information will be collected, shared, and maintained in an independent closet to protect confidentiality before, during, and after the trial. The protocol has been registered at Chinese Clinical Trials Register, (http://www.chictr.org.cn/showproj.aspx?proj=128232, Identifier: ChiCTR2100047946).

## Author contributions

**Conceptualization:** Xiaoliang Chen, Bo Wang, Xiaoling Qian.

**Data curation:** Xiaoliang Chen, Tieyan Wang, Ying Zhu, Jing Han, Jiahe Zhang, Jiali Huang.

**Formal analysis:** Liping Yu, Xiaoliang Chen, Bo Wang, Xiaoling Qian.

**Funding acquisition:** Liping Yu, Xiaoling Qian.

**Investigation:** Xiaoliang Chen, Tieyan Wang, Ying Zhu., Jing Han.

**Methodology:** Bo Wang, Xiaoling Qian.

**Project administration:** Bo Wang, Xiaoliang Chen, Xiaoling Qian.

**Resources:** Xiaoliang Chen.

**Software:** Liping Yu, Ying Zhu.

**Supervision:** Bo Wang, Xiaoling Qian.

**Validation:** Xiaoliang Chen, Bo Wang, Xiaoling Qian.

**Visualization:** Xiaoliang Chen, Xiaoling Qian.

**Writing – original draft**: Xiaoliang Chen, Liping Yu, Bo Wang, Xiaoling Qian.

**Writing – review & editing:** Xiaoliang Chen, Bo Wang, Xiaoling Qian.
